# Mitochondria-derived reactive oxygen species induce over-differentiation of neural stem/progenitor cells after non-cytotoxic cisplatin exposure

**DOI:** 10.3389/fcell.2025.1555153

**Published:** 2025-04-29

**Authors:** Felipe A. Bustamante-Barrientos, Eliana Lara-Barba, Yeimi Herrera-Luna, Cynthia García-Guerrero, Eduardo Silva-Pavez, Jonathan Morales-Reyes, María Jesús Araya, Liliana Yanten-Fuentes, Noymar Luque-Campos, Claudia Altamirano, Ana María Vega-Letter, Patricia Luz-Crawford

**Affiliations:** ^1^ Laboratorio de Inmunología Celular y Molecular, Facultad de Medicina, Universidad de los Andes, Santiago, Chile; ^2^ Centro de Investigación e Innovación Biomédica (CiiB), Universidad de los Andes, Santiago, Chile; ^3^ IMPACT, Center of Interventional Medicine for Precision and Advanced Cellular Therapy, Santiago, Chile; ^4^ Programa de Doctorado en Biomedicina, Facultad de Medicina, Universidad de los Andes, Santiago, Chile; ^5^ Facultad de Odontología y Ciencias de la Rehabilitación, Universidad San Sebastián, Santiago, Chile; ^6^ Escuela de Ingeniería Bioquímica, Pontificia Universidad Católica de Valparaíso, Valparaíso, Chile

**Keywords:** cysplatin, neural stem progenitor cells, mitochondrial ROS, oxidative stress, neurogenesis, stem cell differentiation

## Abstract

**Background:**

Neural stem and progenitor cells (NSPCs) are crucial for nervous system development and self-renewal. However, their properties are sensitive to environmental and chemical factors, including chemotherapy agents like cisplatin, an FDA-approved drug used to treat cancer. Cisplatin inhibits DNA replication but can cause side effects such as nephrotoxicity, ototoxicity, and neurotoxicity. While its cytotoxic effects are well understood, the impact of non-cytotoxic cisplatin concentrations on NSPC differentiation remains unclear.

**Methods:**

This study examined how non-cytotoxic cisplatin exposure influences NSPC differentiation and mitochondrial activity, specifically through reactive oxygen species (ROS) generation. Mitochondrial activity was analyzed via tetrazolium salt (MTT) assay, ATP biosynthesis, mitochondrial membrane potential (ΔΨm), biomass, and ROS production. Glycolytic activity was assessed by extracellular acidification and lactate production. Self-renewal capacity and differentiation were measured using flow cytometry and confocal microscopy. Mitochondrial ROS generation was modulated with Mito-TEMPO.

**Results:**

After 24 h of non-cytotoxic cisplatin exposure (5 μM), mitochondrial activity increased, as shown by higher MTT conversion, ATP content, ΔΨm, biomass, and ROS levels. Despite a stabilization of mitochondrial activity and ROS production by 72 h, this exposure impaired cell cycle progression, self-renewal, and enhanced differentiation toward neuronal and glial lineages. Inhibition of mitochondrial ROS production reduced neuronal and glial differentiation but did not restore self-renewal or cell cycle progression. A decrease in extracellular acidification and lactate production indicated a shift from glycolysis to mitochondrial respiration.

**Discussion:**

Even at subtherapeutic levels, cisplatin disrupts NSPC integrity, driving differentiation through mitochondrial ROS-dependent mechanisms. While inhibiting ROS reduced differentiation, it did not restore NSPC proliferation. These findings highlight the vulnerability of NSPCs to cisplatin, even at doses considered safe. The metabolic shift toward mitochondrial respiration may contribute to this differentiation bias. Future research on co-administration of antioxidant agents during chemotherapy could protect NSPC integrity and mitigate developmental and cognitive risks, especially in neonates exposed via breastfeeding.

## 1 Introduction

Neural stem and progenitor cells (NSPCs) are pivotal for the proper development of the nervous system due to their dual capability to self-renew and differentiate into diverse neural cell types, which collectively contribute to the formation of complex neural circuits (Gotz and Huttner, 2005a). The regulation of these processes is intimately connected to mitochondrial function. Mitochondria, beyond their classical role as cellular energy producers, serve as central hubs for controlling critical cellular processes such as metabolic activity and the generation of reactive oxygen species (ROS) (Khacho et al., 2016).

Specifically, mitochondrial respiration has emerged as a critical regulator of NSPC differentiation, highlighting the importance of mitochondrial integrity in maintaining proper neural development (Khacho et al., 2019). Consequently, NSPCs are highly vulnerable to perturbations in mitochondrial function, particularly those triggered by external agents, including pharmacological compounds that disrupt mitochondrial homeostasis (Bunc et al., 1994; Kleih et al., 2019).

Platinum-based chemotherapy agents, particularly cisplatin, are routinely employed in the treatment of various cancers affecting women of reproductive age, including cervical cancer, triple-negative breast cancer, and pediatric malignancies in adolescents (Hays et al., 2013; Ben-Baruch et al., 1992; de Vries et al., 1989; Lanowska et al., 2011). Cisplatin is valued for its potent ability to target and eradicate rapidly dividing cancer cells (Gandin et al., 2023). However, its therapeutic effectiveness is often accompanied by a range of off-target effects, including nephrotoxicity, ototoxicity, cardiotoxicity, and notably, neurotoxicity (Gandin et al., 2023). In the nervous system, cisplatin’s toxicity manifests through the depletion of NSPCs, leading to reduced brain plasticity and cognitive deficits (English et al., 2020; Boukelmoune et al., 2018).

While the cytotoxic effects of cisplatin on NSPCs are well-established, the consequences of non-cytotoxic exposure remain poorly understood, particularly in contexts where low concentrations of cisplatin, such as those found in umbilical cord blood, amniotic fluid, or breast milk, may still reach the developing nervous system (Lanowska et al., 2011; Egan et al., 1985).

Although cancer during pregnancy is a relatively rare occurrence, it presents a distinctive clinical and ethical challenge. Chemotherapy regimens for pregnant or lactating women pose significant risks for the developing fetus or neonates during lactation (Storgaard et al., 2024). Hence, investigating the biological effects of cisplatin at non-cytotoxic concentrations is fundamental for expanding our understanding of its broader impacts beyond direct cytotoxicity.

In this study, we focus on elucidating the effects of non-cytotoxic cisplatin exposure on mitochondrial fitness and its impact on mitochondrial-driven differentiation processes in neonatal NSPCs. Our results reveal a paradoxical enhancement of mitochondrial function following cisplatin treatment, characterized by elevated oxidoreductase activity, increased mitochondrial membrane potential, enhanced energy production, and intensified ROS generation. These mitochondrial alterations are accompanied by a marked reduction in NSPC proliferation and clonal expansion capacity, alongside a significant increase in differentiation toward neuronal and glial lineages. Intriguingly, pharmacological inhibition of mitochondrial ROS mitigated the aberrant differentiation observed in cisplatin-treated NSPCs, underscoring the role of oxidative stress in driving this process. These findings suggest that mitochondrial oxidative stress plays a critical role in mediating NSPC dysregulation in response to sublethal cisplatin exposure, highlighting the need for further research into protective strategies to mitigate these effects during chemotherapy withdrawal and breastfeeding periods.

## 2 Material and methods

### 2.1 Reagents and antibodies

The reagents used in this study included cisplatin (Merck, 232120), mito-TEMPO (Sigma-Aldrich, SML0737), tetramethylrhodamine methyl ester (TMRM, Thermo Fisher, M20036), nonyl acridine orange (NAO, Thermo Fisher, A1372), MitoSOX (Thermo Fisher, M36006), Annexin V-FITC Apoptosis Detection Kit (Sigma-Aldrich, APOAF), Cell Titer Glo2.0 (Promega, G9241), 3-(4,5-dimethylthiazol-2-yl)-2,5-diphenyltetrazolium bromide (MTT, Sigma-Aldrich, 11465007001), and glycolysis assay (Abcam, ab197244).

The antibodies used included anti-Nestin (Invitrogen, MA1-110), PE-labeled anti-Sox2 (BD Biolegend, 14A6A34), anti-beta-III-tubulin (Novus Biologicals, NB100-1612), anti-doublecortin (Novus Biologicals, NBP1-92684), anti-GFAP (Thermo Fisher, PA1-10004), anti-Aldolase-C (PA5-12317), anti-MAP2 (Abcam, A85363), BUV421-labeled anti-Ki-67 (BD Biosciences, 562899), and anti-BrdU (DSHB, G3G4). Secondary antibodies used were donkey anti-mouse Alexa 488 (Invitrogen, A32766), donkey anti-rabbit Alexa 555 (Invitrogen, A31572), and donkey anti-goat Alexa 637 (Invitrogen, A32849).

### 2.2 Animals

C57BL/6 mice were obtained from The Jackson Laboratory (Bar Harbor, Maine, US) and housed at the animal facilities of Universidad de los Andes, Santiago, Chile. The mice were kept in a 12-h light/dark cycle with a controlled temperature of 24°C. Newborn (PN0) mice were used to isolate NSPCs directly from the telencephalon, as described in Bustamante-Barrientos et al. (2023). All procedures were conducted in agreement with our Institutional Guidelines for Animal Care and were approved by the Scientific and Ethics Committee of Universidad de los Andes (Certificate: CEC2021013).

### 2.3 Cell isolation and culture

NSPCs were dissected directly from the dorsal and lateral telencephalic tissue and cultured in NeuroCult Basal medium supplemented according to the manufacturer instructions (Stem Cell Technologies, 05702), containing 10 ng/mL epidermal growth factor (EGF, Stem Cell Technologies, 78006), 20 ng/mL basic fibroblast growth factor (bFGF, Stem Cell Technologies, 78003), 2 μg/mL heparin (Stem Cell Technologies, 07980), and penicillin-streptomycin (Thermo Fisher, 15140122). Cells were plated at a density of 60,000 cells/mL in non-adherent dishes, incubated at 37°C with 5% CO_2_, and passaged three times before experimental use. Neurospheres were mechanically dissociated before drug treatments or assays.

### 2.4 Differentiation assay

Differentiation assays were conducted as described in Bustamante-Barrientos et al. (2023). Briefly, neurospheres at passage three were dissociated and plated onto poly-l-lysine-coated coverslips in 24-well plates. Neurons were obtained by differentiating NSPCs during five *in vitro* days (5 DIV) in NeuroCult Basal medium containing 1% fetal bovine serum (FBS), while astrocytes were differentiated during 10 days in medium containing 5% FBS and 10 ng/mL EGF. Cultures were refreshed every 48 h, and the lineage commitment was confirmed by immunostaining against: βeta-III-tubulin (β3) and Doublecortin (DCX) for neurons; and the Glial Fibrillary Acid Protein (GFAP) and Aldolase-C (AldoC) for glia cells. Finally, nuclei were counterstained with DAPI.

For image analyses, automatic segmentation of NSPCs-derived MAP2-positive neurons was performed. Briefly, ImageJ Fiji software was programmed to quantify the maximum branch length, number of branches, average length of branches, and intersections between branches. These measurements incorporate local thresholding methods using the Niblack algorithm for cells segmentation.

### 2.5 Immunofluorescence

Primary antibodies were diluted in buffer containing 2.5% BSA and 0.1% Triton X-100. Coverslips were processed for immunofluorescence using anti-Nestin (1:50), PE-labeled anti-Sox2 (1:50), anti-beta-III-tubulin (1:1000), anti-doublecortin (1:200), anti-GFAP (1:1000), anti-MAP2 (1:2500), anti-BrdU (1:50), and anti-aldolase-C (1:250). Samples were imaged using a Leica TCS-SP8 confocal microscope, and cell counts were analyzed with the Fiji open-source software. Image processing was performed to quantify neuronal and astroglial lineages (Figure 6), as well as morphological parameters including maximum branch length, average branch length, number of branches, and intersection points (Supplementary Figure S1).

### 2.6 Oxidoreductase activity and ATP content

For the assessment of oxidoreductase activity, we employed the tetrazolium salt MTT assay to quantify the bioconversion of MTT into formazan. The resulting formazan crystals were solubilized, and the absorbance values were measured using a standard spectrophotometer, providing a quantitative readout of cellular metabolic activity. NSPCs were treated with increasing doses of cisplatin from 1 to 10 μM for 24, 48 and 72 h. Absorbance at 570 nm was measured and results were normalized to untreated controls, as described by Ghasemi et al. (2021).

ATP levels were assessed using the Cell Titer-Glo Luminescent Cell Viability Assay following the manufacturer´s instructions (Promega, G7570). Briefly, third-passage NSPCs were treated with increasing concentrations of cisplatin from 1 to 10 μM and analyzed for luminescence after 10 min. Data were recorded as relative luminescence units (RLU) utilizing a BioTek FLx800 microplate reader, as described in Bustamante-Barriento et al. (Bustamante-Barrientos et al., 2023).

### 2.7 Flow cytometry

Flow cytometry analysis was performed on single NSPCs stained with: Tetramethylrhodamine Methyl Ester (TMRM; Thermo Fisher, T668) and Mito Probe JC-1 (Thermo Fisher, M34152) to measure the mitochondrial membrane potential (ΔΨm); JC-1 monomeric signal and Nonyl Acridine Orange 10-Nonyl Bromide (NAO; Thermo Fisher, A1372) to measure mitochondrial biomass; and MitoSOX (M36008) to measure mitochondrial ROS generation. As well, dying cell populations either in apoptosis or necrosis were measured by Apoptosis Kit Detection (BD Bioscience, 559763) complemented with Propidium Iodide (PI; Thermo Fisher, P1304MP). Moreover, we evaluated cell cycle progression using Ki-67 (Biolegend Pacific Blue, 652422) complemented with PI (Thermo Fisher, P1304MP). The acquisition was assessed with a FACS CANTO II (BD bioscience) and the analysis with FlowJo software (version 10.0.7).

### 2.8 Measurement of glycolytic activity

Extracellular acidification was determined by using a fluorescence microplate reader and a pH-sensitive reagent, following the manufacturer’s instructions (Abcam, ab197244). Lactate levels were quantified using a YSI 2700 automated analyzer.

### 2.9 Statistical analysis

Data were analyzed through one-way ANOVA with Bonferroni´s test using GraphPad Prism software (CA, United States). All the data is presented as mean ± standard deviation (SD). Significance with a p-value <0.05 was considered statistically significant.

## 3 Results

### 3.1 Non-cytotoxic cisplatin exposure enhances mitochondrial function and promotes mitochondrial-derived ROS production in NSPCs

**FIGURE 1:**
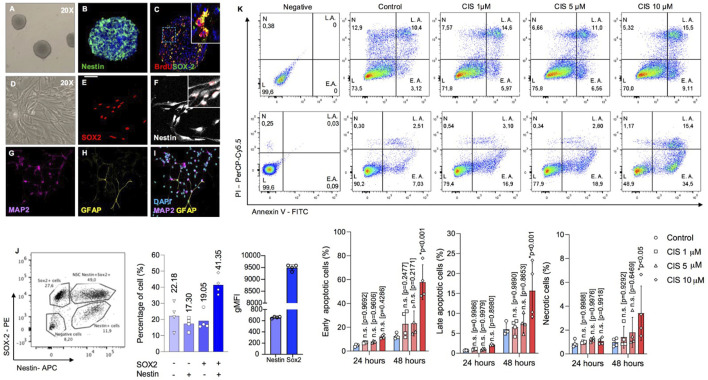
Determination of cisplatin cytotoxicity in neonatal mouse-derived NSPCs. **(A–C)** Representative phase contrast image of 6DIV neurospheres **(A)**, and immunostaining against **(B)** nestin and **(C)** BrdU/SOX2. **(D–F)** Representative phase image of **(D)** 2DIV monolayer NSPCs cultures, and immunostaining against **(E)** SOX2 and **(F)** Nestin. **(G–I)** Representative immunostainings of **(G)** MAP2 and **(H)** GFAP-positive cells after 5 and 10 differentiation days, respectively. **(J)** The abundance of Nestin-SOX2 double-positive cells, corresponding to *bona fide* NSPCs; intermediate progenitor cells (Nestin-negative and SOX2-positive); and postmitotic cells (Nestin-positive and SOX2-negative) in neurospheres was quantified by flow cytometry as a percentage, along with the geometric MFI for SOX2 and Nestin, respectively (n = 4). Nestin-positive cells likely represent a population that has lost its stem-like characteristics and is presumably initiating differentiation. Please note that the Nestin-SOX2 double-negative population in the bars also includes cells that fall outside the defined populations. **(K)** Early and late apoptosis, as well as necrosis, in NSPCs cultured alone or in the presence of 1, five or 10 μM cisplatin, were measured by flow cytometry to assess cytotoxicity at different drug concentrations. The data is presented as the percentage of: Annexin-V (AV)-positive Propidium Iodide (PI)-negative cells (early apoptosis); AV-PI-double positive cells (late apoptosis); and AV-negative PI-positive cells (necrosis) (n = 4). Differences with p < 0.05 were considered significant (one-way ANOVA with Bonferroni´s test). n. s. = no significant differences regarding the control groups without cisplatin treatment at 24 and 48 h, respectively. Scale bars: A, D = 70 μm; B-C, E-I = 35 μm.

Cisplatin is a widely used chemotherapeutic drug, known for its effectiveness in targeting rapidly dividing cells (Gandin et al., 2023). Previous *in vitro* studies have shown that cisplatin induces significant toxicity in nervous cells at micromolar concentrations (English et al., 2020; Boukelmoune et al., 2018). Thus, to evaluate the impact of non-cytotoxic cisplatin concentrations, we conducted a dose-response analysis using 3D neurosphere cultures to work below those concentrations that induce apoptosis in NSPCs (Deleyrolle et al., 2008). Neurospheres exhibited classical biochemical markers of NSPCs, including the expression of Nestin and SOX2, and demonstrated proliferative activity as evidenced by BrdU incorporation (Figures 1A–C). Flow cytometry analysis revealed that approximately 19.9% of the cells in neurospheres were single-positive for Sox2, while 41.35% were double-positive for Nestin and Sox2, confirming that ∼60.4% of the cells exhibited NSPC-like features. In turn, approximately 17.30% of the cells were single-positive for Nestin, likely representing differentiating cells that have lost the expression of the stemness-associated transcription factor SOX2 (Gotz and Huttner, 2005a). Meanwhile, cells negative for both markers, along with those that did not fall within the defined populations, were classified as double-negative, comprising up to 22.18% (Figure 1J). On the other hand, under differentiation conditions such as adherent substrates and serum-enriched media (Pirhajati Mahabadi et al., 2015), these cells can generate MAP2+ neurons and GFAP + glia (Figures 1D–I).

To define the cytotoxic threshold, NSPCs were exposed to increasing concentrations of cisplatin from 1 to 10 μM, for 24 and 48 h (h) (Figure 1K). Neurospheres were mechanically disaggregated and analyzed by flow cytometry using Annexin-V (AV) and propidium iodide (PI) staining. This analysis categorized cells as: healthy (double-negative), early apoptotic (AV+/PI-), late apoptotic (AV+/PI+), or necrotic (AV-/PI+). At a concentration of 10 μM, cisplatin significantly increased the proportion of apoptotic cells after 48 h of exposure, with approximately 50% more cells undergoing early apoptosis (p < 0.0001), a 10% rise in late apoptosis (p < 0.001), and a 2% in necrosis (p < 0.05). In contrast, no statistical changes were observed at concentrations of 1 μM (p = 0.9686) or 5 μM (p = 0.9318) (Figure 1K), which were therefore considered as non-cytotoxic.

**FIGURE 2:**
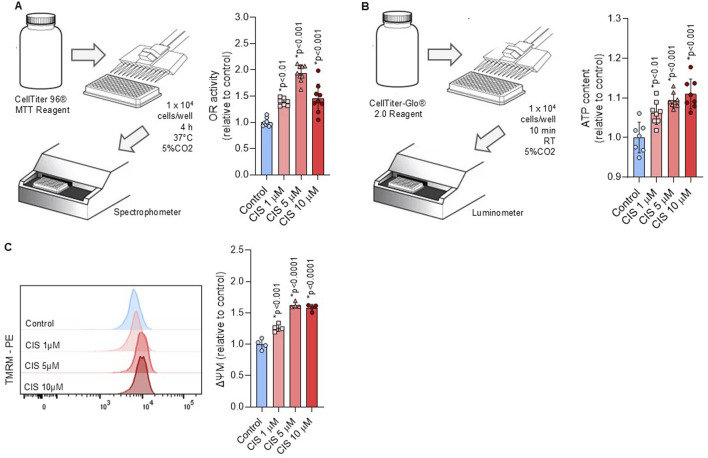
Non-cytotoxic cisplatin exposure boosts mitochondrial activity. **(A,B)** A workflow scheme outlines the procedure to measure the **(A)** oxidoreductase activity via tetrazolium salt conversion (n = 9) and **(B)** ATP content via luminescence (n = 8) in NSPCs cultured alone or in the presence of 1, five or 10 μM cisplatin. The data is presented as mean ± SD. **(C)** Mitochondrial membrane potential (ΔΨM) was measured by flow cytometry using the TMRM staining in NSPCs cultured alone or in the presence of 1, five or 10 μM cisplatin (n = 4). The data is presented as a ratio of the geometric mean fluorescence intensity (MFI) of TMRM staining in cisplatin-treated conditions, expressed relative to the mean of the control group (n = 4). Differences with p < 0.05 were considered significant (one-way ANOVA with Bonferroni´s test).

Platinum-based drugs are known to impair mitochondrial function and promote oxidative stress in target cells (Marullo et al., 2013; Kleih et al., 2019). To assess mitochondrial function, NSPCs were exposed to cisplatin at both cytotoxic and noncytotoxic concentrations. We measured oxidoreductase activity, ATP content, mitochondrial biomass, and membrane potential (Δψm). We showed that cisplatin-treated NSPCs respond with a dose-dependent increase in mitochondrial activity, as measured by their elevated conversion of MTT to formazan (Figure 2A). This finding correlated with a significant increase in both ATP content and Δψm, measured by luminescence and TMRM fluorescence, respectively (Figures 2B, C). Notably, mitochondrial activity and ATP content displayed a biphasic pattern, with suppression at 48 h followed by stabilization at 72 h (Supplementary Figure S2), whereas Δψm stabilize at 48 h (Supplementary Figure S3).

**FIGURE 3:**
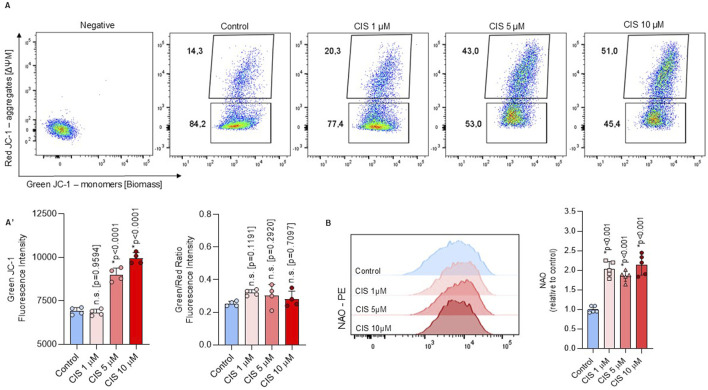
Non-cytotoxic cisplatin exposure increases mitochondrial biomass. **(A, B)** Mitochondrial biomass was measured by flow cytometry through the JC-1 (n = 4) and NAO (n = 5) staining in NSPCs cultured alone or in the presence of 1, five or 10 μM cisplatin. A ratio between JC-1 red aggregates and JC-1 monomers in **(A’)** The data is presented as the MFI in cisplatin-treated conditions, expressed relative to the mean of the control group. Differences with p < 0.05 were considered significant (one-way ANOVA with Bonferroni´s test) as compared to control group.

The non-cytotoxic increase in Δψm, as measured by TMRM, correlates with a significant increase in JC-1 red aggregates, which represent the more polarized mitochondrial fraction. However, after normalization with JC-1 green monomers, no statistical differences were found (Figure 3A). JC-1 monomers and Nonyl Acridine Orange (NAO) have been previously used as mitochondrial biomass indicators (Watanabe et al., 2013; Fu et al., 2008) and showed increased fluorescence intensity after cisplatin exposure (Figures 3A, B). These findings suggest that increase in Δψm, indicated by TMRM and JC-1 red aggregates, reflects an increase in the mitochondrial biomass, given that NAO is a membrane potential-independent dye. Notably, mitochondrial biomass stabilizes at 48 h (Supplementary Figure S3), supporting that non-cytotoxic cisplatin induced short-term alterations in mitochondrial function.

**FIGURE 4:**
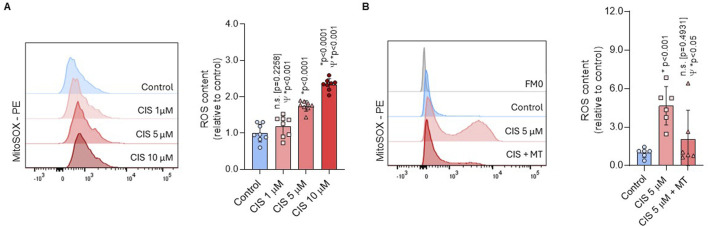
Non-cytotoxic cisplatin exposure boosts mitochondrial ROS production. **(A)** ROS production was assessed in NSPCs using flow cytometry with MitoSOX staining after culturing the cells alone or with 1, 5, or 10 μM cisplatin (n = 8). To corroborate if the ROS originated from mitochondria **(B)** an additional experiment was conducted using 5 μM cisplatin combined with 2.5 μM MitoTEMPO (CIS + MT) (n = 6), a mitochondrial-targeted ROS scavenger. The data is presented as the MFI in cisplatin-treated conditions, expressed relative to the mean of the control group. Differences with p < 0.05 were considered significant (one-way ANOVA with Bonferroni´s test). Statistical comparisons with the control group are indicated with an asterisk (*), while differences against the 5 μM cisplatin condition (CIS) are marked with a Psi symbol (Ψ).

Elevated mitochondrial activity often correlates with increased ROS production (Hamanaka and Chandel, 2010). We showed that ROS generation was significantly higher in NSPCs treated with 5 μM cisplatin compared with both controls (p < 0.0001) and 1 μM cisplatin (p < 0.001) (Figure 4A). Cytotoxic exposure (CIS 10 μM) further increased ROS levels compared to 5 μM cisplatin (p < 0.001). Considering that 1 μM cisplatin did not induce a statistically significant effect on mitochondrial activity, while 10 μM resulted in cytotoxicity, we evaluated the origin of ROS under non-cytosolic conditions using 5 μM cisplatin (Cis-NSPCs). To corroborate whether cisplatin-induced ROS were originated via mitochondria, Cis-NSPCs were co-treated with 2.5 μM Mito-TEMPO (Cis/Mt-NSPCs), which is a mitochondria-targeted antioxidant. We observed that Cis/Mt-NSPCs presented significantly lower ROS levels compared to Cis-NSPCs (p < 0.05), while no differences were observed compared to controls (p = 0.4931) (Figure 4B). This supports the hypothesis that cisplatin-induced ROS are generated via mitochondria without initiating apoptosis signaling. Furthermore, ROS levels showed to be stabilized after 48 h (Supplementary Figure S3), supporting that its overproduction is restricted to the first 24 h.

Lastly, we evaluated extracellular acidification using a pH-sensitive reagent to quantify glycolytic activity during the first 120 min of exposure to non-cytotoxic cisplatin, while lactate production was assessed as an indicator over a 96-h period (Supplementary Figure S4). Our findings indicate that cisplatin induces a long-term suppression of glycolysis, supporting the notion that the enhancement of mitochondrial function reflects a metabolic shift from a glycolytic state.

### 3.2 Cisplatin-induced cell cycle arrest occurs independently of mitochondrial ROS generation

**FIGURE 5:**
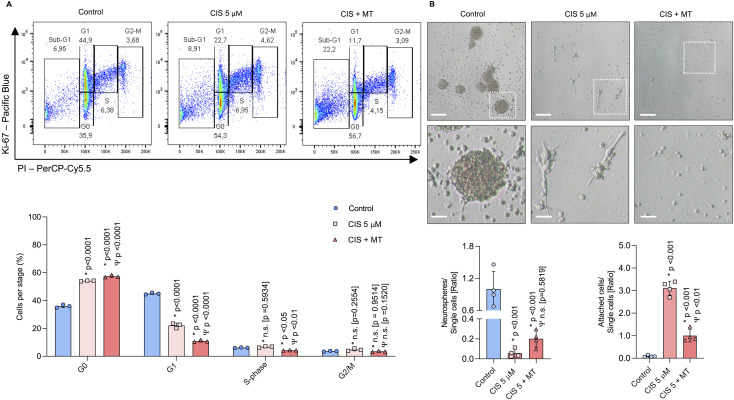
Cisplatin-treated NSPCs undergo cell cycle arrest and lose their self-renewal capacity in a ROS-independent manner. **(A)** Cell cycle progression in NSPCs was evaluated by flow cytometry using Ki-67 and PI staining to separate into three categories: non-dividing, DNA-replicating, and dividing cells. The results are presented as the percentage of NSPCs in each cell cycle phase: G0, G1, S, and G2/M (n = 3). **(B)** Representative phase-contrast images of NSPCs cultured for 6 days under proliferative and self-renewal conditions. The number of neurospheres and attached cells were normalized by the number of single cells and expressed as ratios respectively (n = 4). Statistical significance was set at p < 0.05 (one-way ANOVA with Bonferroni’s test). Comparisons with the control group are indicated by an asterisk (*), while differences relative to the CIS group are marked with a Psi symbol (Ψ). Scale bars: 125 μm; magnification frame: 32 μm.

To assess whether cisplatin-induced ROS affects cell cycle progression, we analyzed the distribution of NSPCs across different cell cycle phases using Ki-67 and propidium iodide (PI) staining. Ki-67 is a proliferation marker expressed in all active cell cycle phases (G1, S, G2, and M) but absent in G0, allowing the distinction between proliferating and quiescent cells. Meanwhile, PI binds to DNA, and its fluorescence intensity enables the differentiation of cells in G1, S, and G2/M phases based on DNA content (Rahme, 2021). Cisplatin-treated NSPCs (5 μM) exhibited a significant G0-phase arrest (54.16% ± 0.23; p < 0.0001) compared to controls (35.96% ± 1.1), while G1-phase entry was reduced by 22.9% (p < 0.0001) (Figure 5A). Co-treatment with Mito-TEMPO did not alleviate G0 arrest but instead exacerbated it (p < 0.0001). There were no significant differences in S-phase or mitotic NSPCs treated with cisplatin alone. However, cells co-treated with Mito-TEMPO (Cis/Mt) showed reduced S-phase entry compared to both controls (p < 0.05) and cisplatin alone (p < 0.01). These findings suggest that baseline mitochondrial ROS is necessary for proper cell cycle progression, while excessive ROS promotes cell cycle arrest.

### 3.3 Cisplatin-induced impairment of self-renewal occurs independently of mitochondrial ROS generation

Self-renewal capacity was assessed by counting the number of neurospheres formed from single NSPCs cultured for 5 days *in vitro* (DIV) (Nagao et al., 2008; Han et al., 2017). The ratios of neurospheres and attached cells were normalized to the number of single cells per field. Cis-NSPCs exhibited a significant reduction in neurospheres formation (p < 0.01) and an increase in attached cells (p < 0.01) compared to controls (Figure 5B). Inhibition of mitochondrial ROS did not restore the formation of neurospheres: however, Cis/Mt-NSPCs adhered significantly less (p < 0.01), suggesting that mitochondrial ROS may promote differentiation and thereby reduce self-renewal capacity.

### 3.4 Non-cytotoxic cisplatin exposure drives differentiation through mitochondrial ROS

**FIGURE 6:**
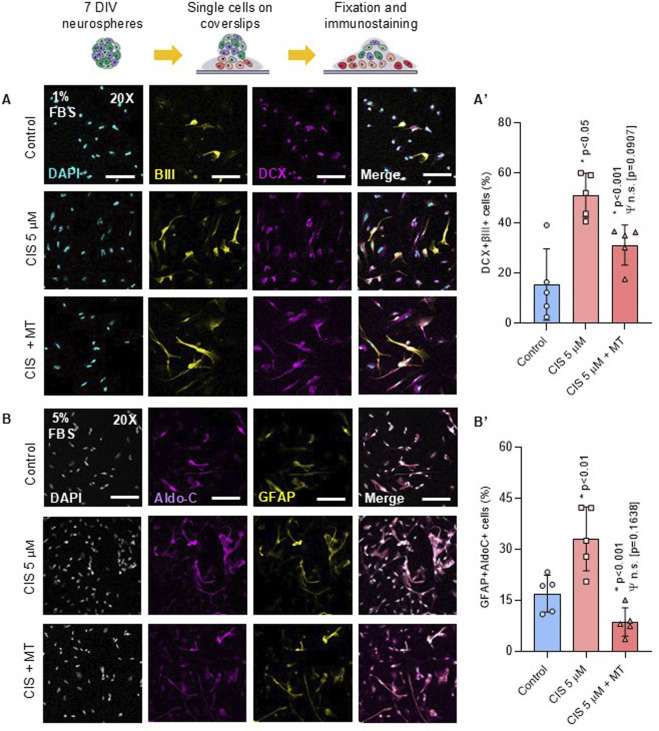
Cisplatin-treated NSPCs promotes NSPCs differentiation into neurons and astrocytes. NSPCs were seeded into poly-l-lysine-coated coverslips for 5 and 10 days to differentiate into neurons and astrocytes, respectively **(A, B)** Representative immunofluorescence against βIII-tubulin (βIII), Doublecortin (DCX), GFAP and Aldolase C (AldoC). **(A′, B′)** Quantification of βIII-, DCX-, GFAP- and AldoC-positive cells. The data is presented as the percentage of positive cells in the total number of cells (nuclei) (n = 5). Differences with p < 0.05 were considered significant (one-way ANOVA with Bonferroni´s test). Statistical comparisons with the control group are indicated with an asterisk (*), while differences against the CIS group are marked with a Psi symbol (Ψ). Scale Bars: 35 μm.

Non-cytotoxic exposure to cisplatin enhanced neurogenic commitment, as evidenced by a 35.66% increase in the generation of βIII-DCX-positive neurons compared to controls (p < 0.001) (Figure 6A). Additionally, the number of GFAP-AldoC-positive cells increased up to 16.13% (p < 0.01) (Figure 6B), indicating that NSPCs were in a state of non-preferential overdifferentiation, a condition often associated with premature NSPCs depletion (Lazutkin et al., 2019). Cis/Mt-NSPCs showed a partial reverse in the uncontrolled generation of neurons and glia, entailing a 19.93% (p < 0.05) and 24% (p < 0.001) reduction in the percentage of βIII-DCX-positive cells and GFAP-AldoC-positive cells, respectively. No significant differences were observed in neuronal maturation after 15 DIV, as assessed by measuring the number, length, and intersections among MAP2-positive neurons (Supplementary Figure S1).

**FIGURE 7:**
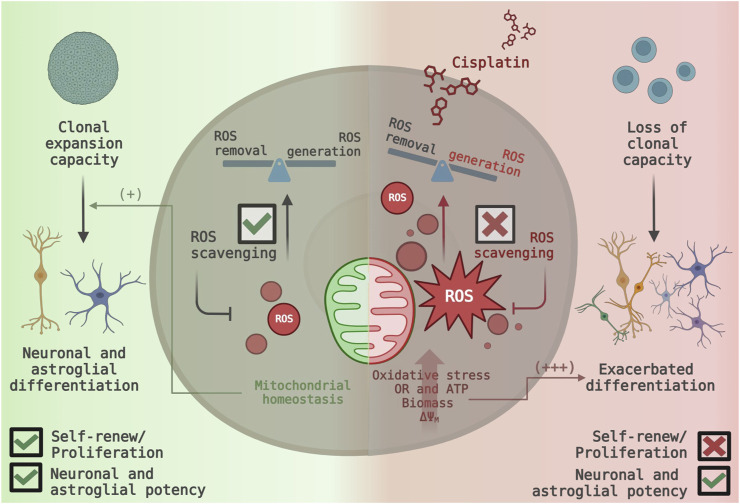
Proposed model of cisplatin-induced mitochondrial ROS generation and its functional consequences in NSPC fate. (Left Panel) Under normal conditions, NSPCs maintain their proliferative activity and self-renewal capacity. Efficient ROS scavenging ensures mitochondrial homeostasis, leading to a balance between ROS generation and removal (Right Panel) Non-cytotoxic cisplatin exposure disrupts mitochondrial function, increasing ROS generation and overwhelming scavenging systems. The resulting oxidative stress causes a dramatic shift towards neuronal and astrocytic differentiation, while indirectly inhibiting cell cycle progression and leading to the loss of self-renewal capacity.

In summary, non-cytotoxic concentrations of cisplatin enhanced mitochondrial function and promoted the generation of mitochondrial-derived ROS in NSPCs, leading to increased oxidative stress. This increase in mitochondrial ROS production was accompanied by significant cell cycle arrest in the G0-phase, reduced self-renewal capacity, and an accelerated shift toward neurogenic and astrocytic differentiation. Interestingly, inhibiting mitochondrial ROS did not restore cell cycle progression or neurospheres formation but did reduce the proportion of cells committed to differentiation (Figure 7). These findings emphasize the impact of cisplatin on NSPCs biology beyond its cytotoxic effect.

## 4 Discussion

Neonatal neural stem and progenitor cells (NSPCs) retain the ability to self-renew and differentiate into neurons and macroglial cells, a balance regulated by intrinsic and extrinsic factors (Gotz and Huttner, 2005b; Rakic, 2000). Given their essential function of NSPCs in neurodevelopmental plasticity and neural homeostasis, disturbances in the NSPCs’ biology could have significant implications, particularly for vulnerable populations such as neonates and infants, whose nervous system are still developing (Zhang et al., 2019). Our study proves that non-cytotoxic exposure to cisplatin, a widely used chemotherapeutic agent, increases mitochondrial activity and ROS production, promoting differentiation into neurons and astrocytes. Meanwhile, the proliferative and self-renewal capacity were suppressed independently of ROS (Figure 7).

Cisplatin is well-known for its cytotoxic effects causing DNA damage, mitochondrial dysfunction, and oxidative stress (Boukelmoune et al., 2018; Lomeli et al., 2017). Literature often associates these effects with enhanced cellular sensitivity to chemotherapy (Kleih et al., 2019). Cisplatin´s bioaccumulation induces mitochondrial damage and reduces NSPCs survival in the hippocampus, as well as modest neuronal dendritic branching and spine density, thereby compromising brain plasticity and cognitive function (Ferreira et al., 2016; Lomeli et al., 2017; Boukelmoune et al., 2018). Our results show a paradoxical increase in mitochondrial function and ROS generation. This builds on prior works by Scalco-Ferreira et al., who demonstrated that non-cytotoxic cisplatin can alters axonal and neurite outgrowth (Ferreira et al., 2016). Although authors did not report findings regarding alterations in mitochondrial biology, our results complement this evidence as non-cytotoxic cisplatin induces overdifferentiation of NSPCs via mitochondrial-ROS generation.

Other chemotherapeutic agents have been shown to disrupt mitochondrial metabolism, impacting both the central and peripheral nervous systems. Notable examples include anti-tubulin compounds like paclitaxel and vincristine, proteasome inhibitors such as bortezomib, and other platinum-based compounds like oxaliplatin. These drugs are known to disturb mitochondrial calcium homeostasis, increase membrane permeability, impair ATP production, and trigger mitochondria-mediated apoptosis through Cyt-c release and Bcl-2 inactivation (Kidd et al., 2002; Andre et al., 2000; Zheng et al., 2011; Tari et al., 1986; Landowski et al., 2005; Joseph and Levine, 2006; Zheng et al., 2012; Xiao and Bennett, 2008; Arango et al., 2004). Moreover, both paclitaxel and doxorubicin exposure showed to alter other cellular (non-neural) processes like the immune response without overt cytotoxicity (Kaneno et al., 2011; Shurin et al., 2009).

We observed that cisplatin treatment increased mitochondrial oxidoreductase activity, ATP production, mitochondrial biomass, and membrane potential, all indicative of heightened mitochondrial function (Rodenburg, 2011; Sharapova et al., 2024). This hypothesis is supported by Kleih et al., who proved that mitochondrial constituents and respiration are upregulated when cells are exposed to cytotoxic agents (Kleih et al., 2019). The simultaneous increase in mitochondrial biomass supports the idea of cellular compensation in response to bioenergetic stress. Given that platinum-based drugs inhibit upstream glycolytic pathways (Shiratori et al., 2019; Tan et al., 2022), the observed increase in mitochondrial activity may represent a protective mechanism in NSPCs transitioning from a predominantly glycolytic metabolism (Khacho et al., 2019; Deldar et al., 2018). Interestingly, our data aligns with this finding, as NSPCs show a rapid decrease in glycolytic activity upon exposure to non-cytotoxic cisplatin.

The observed mitochondrial hyperactivity was accompanied by a marked rise in mitochondrial-derived ROS levels. ROS have long been recognized as metabolic byproducts, but they also play essential roles in regulating processes such as proliferation, differentiation, and autophagy (Checa and Aran, 2020). Cisplatin-induced ROS production has been extensively documented, with ROS implicated in mitochondrial and nuclear DNA damage, bioenergetic dysfunction, and apoptosis (Marullo et al., 2013; Kleih et al., 2019; Xue et al., 2020; Boukelmoune et al., 2018). Boukelmoune et al. reported that cisplatin reduces the number of DCX-positive neuroblasts and mitochondrial respiration in adult NSPCs, leading to their depletion (Boukelmoune et al., 2018). However, our findings differ significantly, as embryonic and neonatal NSPCs demonstrate greater resilience to chemical and environmental insults than adult NSPCs (Colombo et al., 2006; Barazzuol and Jeggo, 2016). Additionally, we used a longer cisplatin exposure period (24 h vs 8 h), potentially allowing for mitochondrial damage repair. Nevertheless, the precise mechanisms underlying ROS accumulation in our model remain unclear, as ROS production can result from either mitochondrial damage or increased mitochondrial activity (Hernansanz-Agustin and Enriquez, 2021).

Inhibiting mitochondrial ROS generation partially reversed the cisplatin-induced over-differentiation toward neuronal and astroglial lineages, suggesting that mitochondrial activity plays a critical role in this process. Mechanistically, ROS act as upstream regulators of various signaling pathways, either at the transcriptional or post-translational level. One of the most relevant post-translational modifications is cysteine oxidation, which can lead to sulfenylation (-SOH), disulfide bond formation (-S-S-), sulfinylation (-SO_2_H), or sulfonylation (-SO_3_H), depending on the level of oxidative stress (Garrido Ruiz et al., 2022). These modifications function as redox switches that regulate the activity of key proteins in cellular signaling, including AMPK, PI3K/Akt/mTOR, MAPK/ERK, Wnt/β-catenin, and Nrf2 (Le Belle et al., 2011; Bustamante-Barrientos et al., 2023; Kasai et al., 2020; Haack et al., 2015; Tsai et al., 2021; Zhang et al., 2022). These pathways integrate metabolic cues to regulate neurogenic differentiation, and excessive mitochondrial ROS can disrupt this balance, pushing NSPCs out of the proliferative state prematurely and favoring differentiation over self-renewal.

Additionally, glycolysis and its byproducts serve as critical checkpoints for maintaining NSPCs in an undifferentiated state (Folmes and Terzic, 2016; Dam and Caruso, 2012; Khacho et al., 2019). A sudden shift toward mitochondrial respiration could further accelerate the loss of proliferative and self-renewal capacity by redirecting metabolic flux away from pathways that sustain stemness. While mitochondrial respiration efficiently generates ATP through oxidative phosphorylation, it does so at the expense of anabolic processes required for cell division, reinforcing the transition toward differentiation (Spinelli and Haigis, 2018). In contrast, glycolysis not only provides ATP but also supplies essential biosynthetic intermediates necessary for rapid cell proliferation. Our results align with this notion, as the abrupt shift toward mitochondrial metabolism occurred at the expense of glycolytic activity. Given that oxidative stress has been reported to promote NSPC differentiation (Huang et al., 2012; Eltutan et al., 2022; Walton et al., 2012), we propose that cisplatin-induced mitochondrial hyperactivity disrupts the balance between proliferation and differentiation by exceeding a critical redox threshold. This metabolic imbalance may drive cells prematurely out of the proliferative cycle and into differentiation, reinforcing the link between mitochondrial activity, cell cycle regulation, and fate commitment.

Though the role of mitochondrial ROS in astrocytic differentiation has not been extensively studied, Schneider et al. found that NSPCs with DNA damage favored astrocytic differentiation over self-renewal (Schneider, 2014). Given cisplatin’s capacity to induce both nuclear and mitochondrial DNA damage, it is plausible that alternative pathways may drive astrocytic differentiation, but further validation is needed. Additionally, cisplatin-induced ROS led to G0-phase cell cycle arrest, reducing NSPC self-renewal capacity. This reduction in cycling cells likely reflects cisplatin’s action mechanism, which involves DNA replication interference (Dasari et al., 2022). Delving deeper into the regulation of the repairing machinery could provide novel insights, as it is critical in stem cells maintenance (Mani et al., 2020). The ROS-independent impact on self-renewal suggests an irreversible transition toward cell differentiation. The correlative reduction in the number of neurospheres, alongside the increasing number of attached cells in Cis-NSPCs, supports the existence of a robust transition toward differentiation even when cultured under self-renewal conditions. Hence, it is expected that a depletion of multipotent NSPCs had occurred.

In conclusion, mitochondrial overactivation and elevated ROS levels drive NSPCs’ hyperdifferentiation and disrupt the balance between self-renewal and differentiation in NSPCs. Mitochondria-targeted antioxidants significantly reverse neuronal and astroglia over-differentiation but fail to restore proliferative and self-renewal capacity (Figure 7). Compared to other chemotherapeutics, cisplatin appears to impose an additional layer of mitochondrial stress by promoting excessive ROS production. Previous findings have shown this effect on cytotoxic concentrations (Marullo et al., 2013; Pan et al., 2009), however, our study proves that ROS overproduction also occurs within a non-cytotoxic range.

## 5 Conclusions and perspectives

Our findings provide the first evidence of the impact of low-level cisplatin exposure on neonatal NSPCs, highlighting critical clinical implications. Given that platinum-based compounds are excreted through breast milk, mothers undergoing antineoplastic therapy should be advised against breastfeeding, particularly when treated with alkylating agents like cisplatin (Pistilli et al., 2013). Additionally, cisplatin remains a cornerstone in treating various pediatric cancers, including medulloblastoma, neuroblastoma, and tumors in the chest or abdominal cavity (Saeki et al., 2024; Hartmann et al., 1988; Pritchard et al., 2000). Our data suggest that cisplatin exposure may influence the transition from perinatal NSCs to adult neurogenic niches, potentially disrupting neurodevelopment through mitochondrial overactivation and oxidative stress. This disruption could lead to premature depletion of the NSC pool, impaired neuronal differentiation, and long-term deficits in brain plasticity and cognitive processes (Khacho et al., 2016; Khacho et al., 2019). Understanding these mechanisms is essential for designing neuroprotective strategies to mitigate cisplatin-induced neurotoxicity.

Despite these insights, several limitations must be recognized. Our experiments were conducted *in vitro*, which does not entirely replicate the intricate *in vivo* microenvironment where NSPCs interact with the vasculature, glial cells, and regulatory signaling gradients (Karakatsani et al., 2023; Falk and Gotz, 2017). Furthermore, systemic factors such as immune responses and the blood-brain barrier (Carpentier and Palmer, 2009; Genet et al., 2023) were not accounted for, potentially restricting the translation of our findings to physiological conditions. The lack of standardized frameworks for investigating the non-cytotoxic effects of chemotherapeutics in animal models poses an additional challenge. Future studies must establish protocols for assessing non-cytotoxic chemobrain effects and validating our findings *in vivo*, ultimately leading to the identification of therapeutic interventions, such as the co-administration of mitochondria-targeted antioxidants.

Preclinical studies suggest that antioxidants possess neuroprotective properties and may enhance cognitive performance (Franzoni et al., 2021; Valotto Neto et al., 2024). However, their clinical application remains challenging due to concerns regarding dosing, bioavailability, and potential interactions with chemotherapy (D'Andrea, 2005). Optimizing antioxidants that specifically target mitochondrial oxidative stress holds promise for mitigating cisplatin’s neurotoxic effects without compromising its anticancer efficacy. Future research should explore the precise mechanisms by which ROS drive NSPC over-differentiation, whether through transcriptional regulation, post-translational modifications, or cysteine oxidation-mediated signaling. Additionally, the potential role of cisplatin-induced metabolic shifts from anaerobic to aerobic metabolism remains an unexplored yet intriguing mechanism. Given that glycolytic flux and its byproducts govern NSPC maintenance in an undifferentiated state (Folmes and Terzic, 2016; Dam and Caruso, 2012; Khacho et al., 2019), further investigation into these pathways is crucial for preserving brain homeostasis during cisplatin-based chemotherapy.

## Data Availability

The original contributions presented in the study are included in the article/Supplementary Material, further inquiries can be directed to the corresponding authors.

## Supplementary material

SUPPLEMENTARY FIGURE S1
Non-cytotoxic cisplatin exposure does not impair neuronal maturation. **(A)** An automatic segmentation on NSPCs-derived MAP2-positive neurons was performed. **(B)** ImageJ Fiji software was programmed to quantify the maximum branch length, number of branches, average length of branches and intersections between branches. A total of eight pictures were taken per condition and the average value was graphed. The data is presented as mean ± SD (n = 4). No statistical differences were found between controls and cisplatin-treated NSPCs (p > 0.05; Student’s t-test). Scale bar: 50 μm.

SUPPLEMENTARY FIGURE S2
Cisplatin induces a biphasic response in mitochondrial activity and ATP production, which stabilizes to baseline levels after 72 h **(A)** Oxidoreductase activity via tetrazolium salt conversion (n = 6) and **(B)** ATP content via luminescence (n = 6) in NSPCs cultured alone or in the presence of 1, 5 or 10 μM cisplatin. The data is presented as mean ± SD. Differences with p < 0.05 were considered significant (one-way ANOVA with Bonferroni´s test).

SUPPLEMENTARY FIGURE S3
Mitochondrial membrane potential, biomass and ROS production stabilizes to baseline levels after 48 h. **(A)** Mitochondrial membrane potential (ΔΨM), **(B)** biomass, and **(C)** ROS production, were measured by flow cytometry using the TMRM staining in NSPCs cultured alone or in the presence of 1, 5 or 10 μM cisplatin (n = 6). The data is presented as a ratio of the geometric mean fluorescence intensity (MFI) of TMRM staining in cisplatin-treated conditions, expressed relative to the mean of the control group (n = 4). Differences with p < 0.05 were considered significant (one-way ANOVA with Bonferroni´s test).

SUPPLEMENTARY FIGURE S4
Cisplatin exposure reduces glycolytic activity in NSPCs. **(A, C)** Representative workflow schemes for determining **(B)** extracellular acidification (n = 4) and **(D)** lactate production (n = 6) in NSPCs cultured alone or in the presence of 1, 5 or 10 μM cisplatin. The data is presented as mean ± SD. Differences with p < 0.05 were considered significant (one-way ANOVA with Bonferroni´s test).

SUPPLEMENTARY FIGURE S5
Gating strategy sequence. Representative gating strategy for neurosphere-derived cells, illustrating the sequential subgating procedure applied to identify and isolate viable cells. Initially, cells were selected based on their morphometric characteristics, as determined by the SSC-A and FSC-A parameters. Doublets were excluded by selecting events aligned along the central diagonal in the FSC-A vs FSC-H plot. Dead cells were removed by selecting the population negative for the viability marker, accounting for 89.3% of the total cells. Following this selection, cells were immunolabeled according to the specific requirements of each experiment.
