# Supplementary figures and images for: Mitochondria-derived reactive oxygen species induce over-differentiation of neural stem/progenitor cells after non-cytotoxic cisplatin exposure
Source: Front Cell Dev Biol. 2025 Apr 29;13:1555153. doi: 10.3389/fcell.2025.1555153 (PMC12069383; doi:10.3389/fcell.2025.1555153)

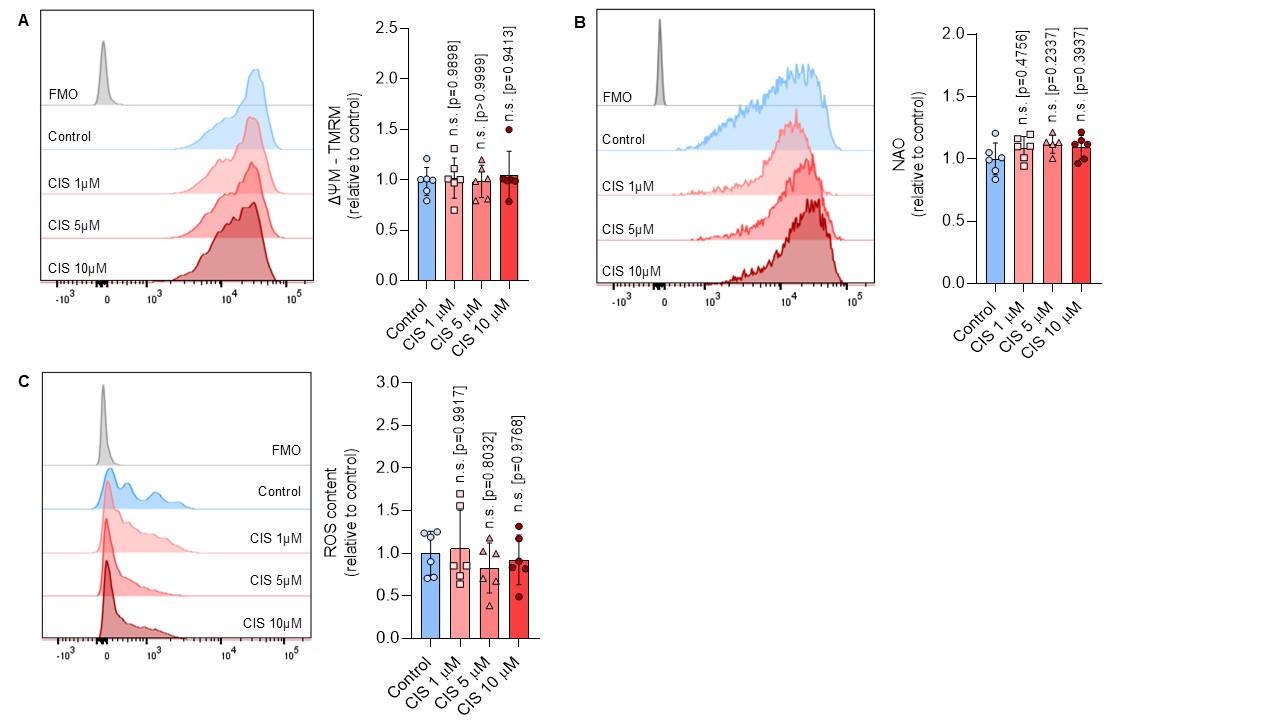

Supplement: Supplementary file 1 [file Image3.jpeg]

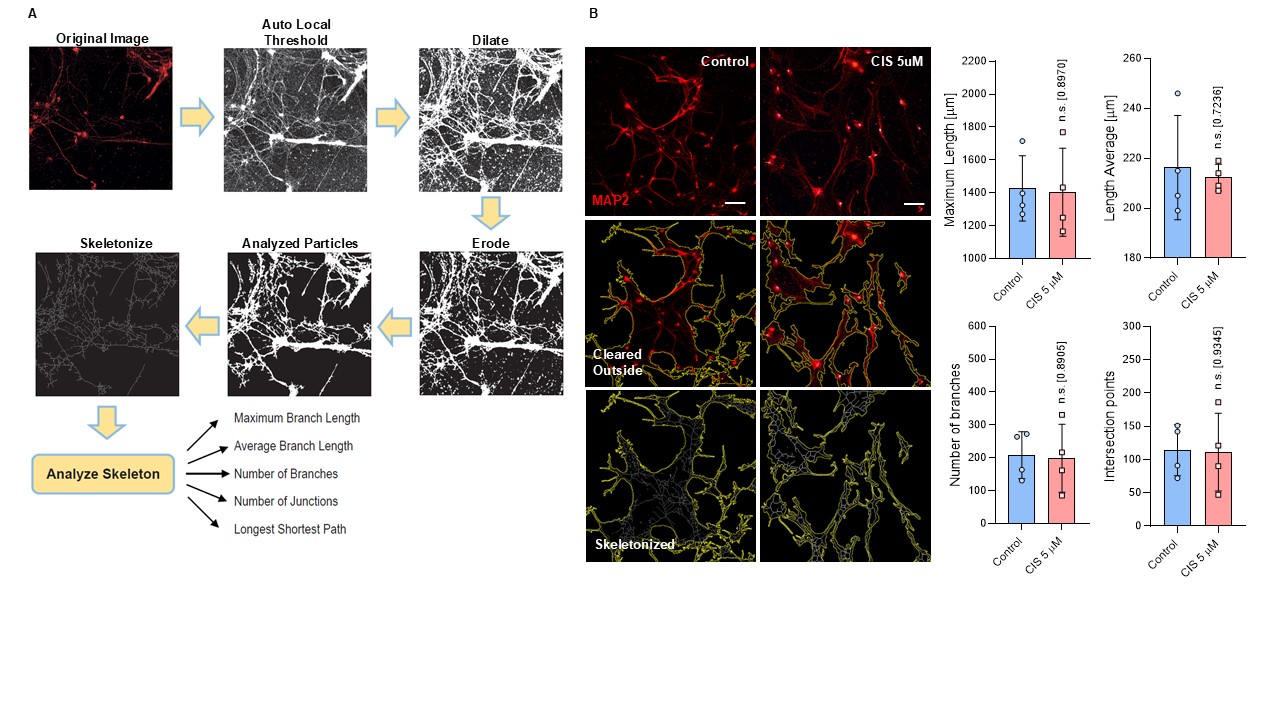

Supplement: Supplementary file 2 [file Image1.jpeg]

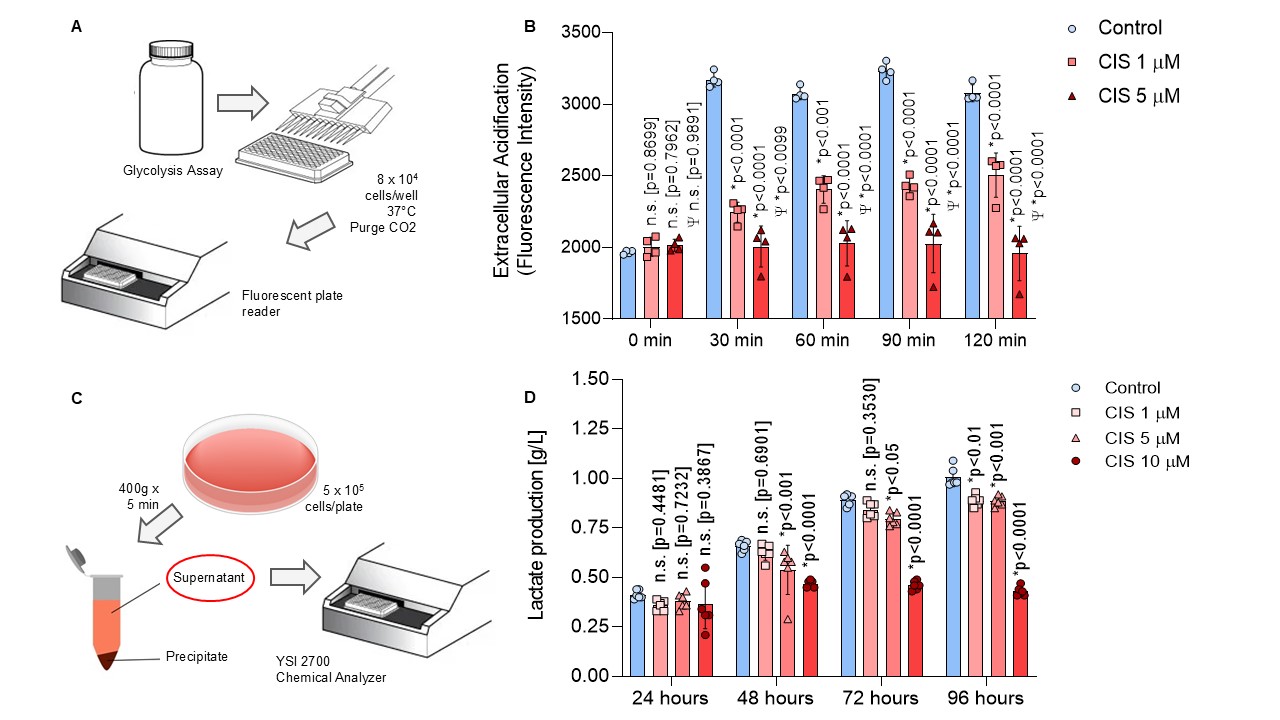

Supplement: Supplementary file 3 [file Image4.jpeg]

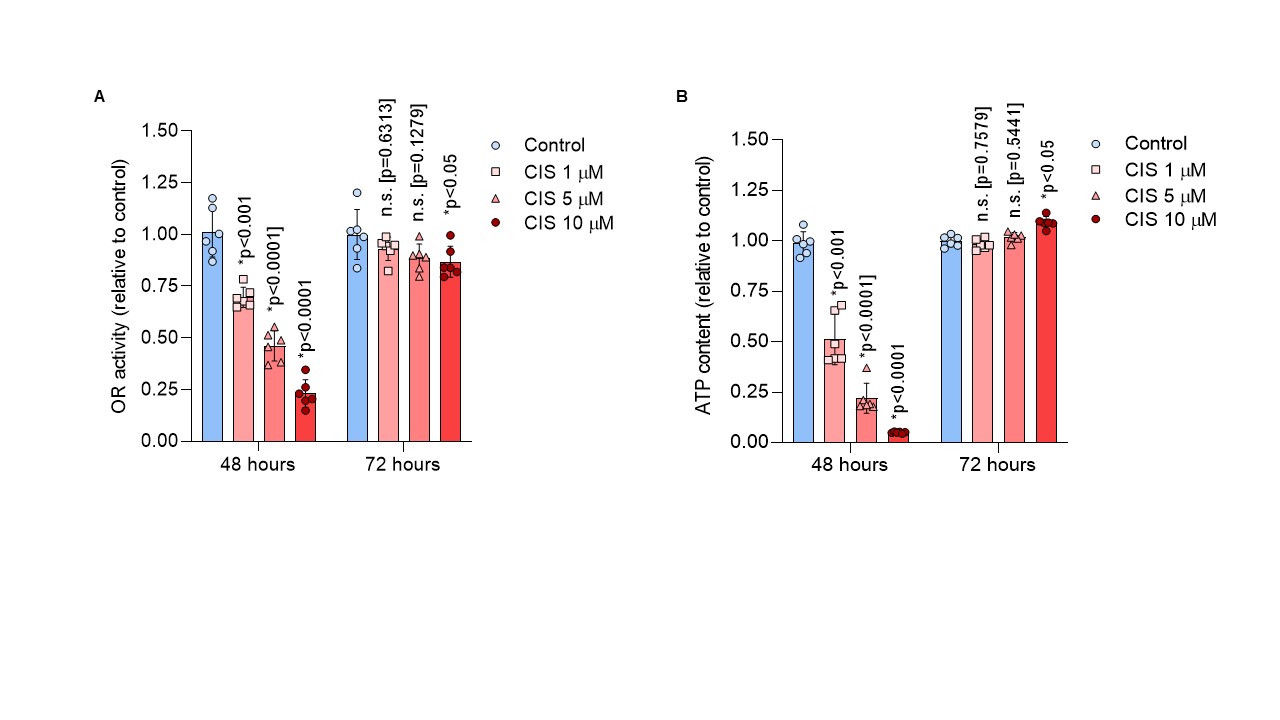

Supplement: Supplementary file 4 [file Image2.jpeg]

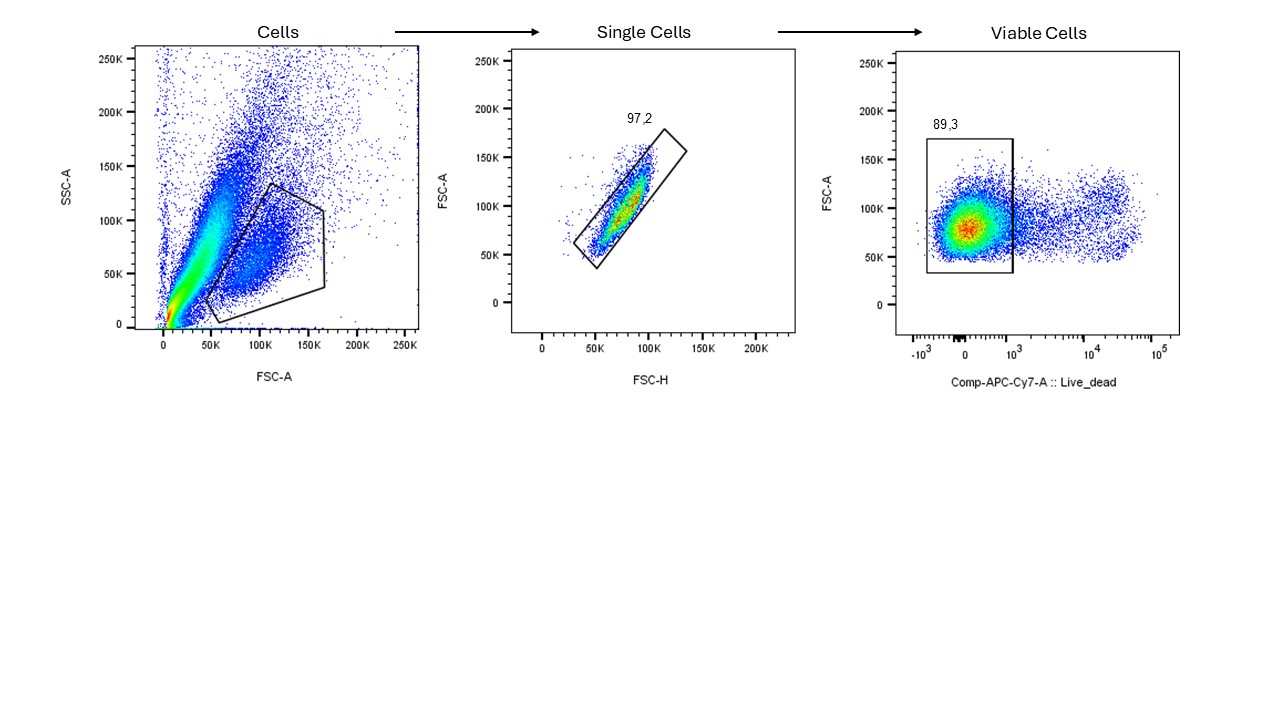

Supplement: Supplementary file 5 [file Image5.jpeg]
